# Impact of hearing loss on cognitive function in community-dwelling older adults: serial mediation of self-rated health and depressive anxiety symptoms

**DOI:** 10.3389/fnagi.2023.1297622

**Published:** 2023-12-14

**Authors:** Fenghui Chen, Yingying Chen, Xin Jiang, Xiaoyang Li, Hongting Ning, Mingyue Hu, Wenxin Jiang, Nan Zhang, Hui Feng, Ping Yan

**Affiliations:** ^1^Xiangya Nursing School, Central South University, Changsha, China; ^2^Nursing School, Xinjiang Medical University, Urumqi, China

**Keywords:** hearing loss, self-rated health, anxiety, depression, cognitive function

## Abstract

**Background:**

Hearing loss can exacerbate cognitive decline; therefore, exploring the mechanisms through which hearing loss affects cognitive function is crucial. The current study aimed to investigate the impact of hearing loss on cognitive function and the mediating role played by self-rated health and depressive anxiety symptoms.

**Methods:**

Using stratified whole-group random sampling, the study employed a cross-sectional design and included 624 participants aged ≥65 years from three communities in Urumqi, China. Cognitive function was assessed using the Mini-Mental State Examination. Hearing function and self-rated health were determined by self-report. The 15-item Geriatric Depression Scale and the 7-item Generalized Anxiety Disorder Scale were used to assess depressive anxiety symptoms. Serial mediation analysis was performed using AMOS 26.0.

**Results:**

Hearing loss can not only negatively affect cognitive function in older adults directly (direct effect = −0.106; SE = 0.045; 95% confidence interval (CI): −0.201 to −0.016), but also indirectly affect the relationship between hearing loss and cognitive function through self-rated health and depressive anxiety symptoms. The results of the serial mediation analysis showed that the total indirect effect of self-rated health and depressive anxiety symptoms was −0.115 (95% CI: −0.168 to −0.070), and the total effect of the model was −0.221 (95% CI: −0.307 to −0.132), with the total indirect effect accounting for 52.04% of the total effect of the model.

**Conclusion:**

Our study discovered that there is a partial mediation of the relationship between hearing loss and cognitive function by self-rated health and depressive anxiety symptoms. It is suggested that by enhancing self-rated health and ensuring good mental health, the decline in cognitive function among older adults with hearing loss can be delayed.

## 1 Introduction

Mild cognitive impairment (MCI) is regarded as the subclinical stage of dementia, and about 10–15% of patients with MCI develop dementia each year (Petersen et al., 2001). According to the World Health Organization (WHO), over 55 million individuals globally suffer from dementia, with approximately 10 million newly diagnosed cases annually, which presents a huge challenge to the long-term care and healthcare system worldwide (WHO, 2023). However, most people with MCI do not necessarily develop dementia. Several studies have found that MCI is reversible, which can restore normal cognitive function. A meta-analysis of 17 studies reported a general reversal that was 27.57% in MCI patients (Xue et al., 2019). Therefore, considering the rapidly aging global population, it is clinically meaningful to search for risk factors associated with cognitive decline, which may help to formulate precise preventive strategies to protect cognitive function and delay the progression of MCI to dementia.

Hearing loss is a sensory impairment that is highly prevalent in the elderly population. The Global Burden of Disease (GBD) Study 2019 showed that hearing loss, as the third disabling factor globally, has affected 1.57 billion people worldwide (GBD 2019 Hearing Loss Collaborators, 2021). Long-term hearing loss interferes with communication and social interaction activities, triggers loneliness and social isolation (Shukla et al., 2020), and has been linked to an increased risk for MCI and dementia (Vassilaki et al., 2019). Over the past few years, there has been significant research conducted on the impact of hearing loss on cognitive abilities, yielding varying outcomes. In the Rancho Bernard Study (RBS), the severity of hearing loss was strongly related to a reduction in overall cognitive and executive function throughout a 24-year follow-up period, and the rate of decline increased over time (Alattar et al., 2020), while in the Blue Mountain Eye Study, cognitive function was not significantly affected by hearing loss (Hong et al., 2016). Although the evidence is inconsistent, hearing loss has been widely recognized as a modifiable risk factor that causes cognitive impairment (Livingston et al., 2020). Notably, the mechanism of action between the two has yet to be completely elucidated. As a result, the current study endeavored to investigate the association between hearing loss and cognitive function as well as identify other modifiable risk factors.

Self-rated health (SRH) is an individual’s subjective assessment based on objective health status (Jylhä, 2009). SRH has been proven to accurately reflect the health status of individuals, and can also predict health-related outcomes such as the decline in physical function, the incidence of chronic diseases, and the mortality of the elderly (Latham and Peek, 2013; Brenowitz et al., 2014; Dramé et al., 2023). Although research on how hearing loss affects SRH in older adults is limited, findings from empirical studies demonstrate that older adults with hearing loss are likely to rate their health as poorer. A recent study conducted in Brazil found that 50.3% of patients with hearing loss reported poor SRH (Anderle et al., 2023). Participating in community events, such as club activities and outings with friends, is extremely important for the physical and social engagement of many older adults. Unfortunately, hearing loss often hinders their ability to participate in these activities (Arnadottir et al., 2011). Hearing loss reduces their involvement in social settings, leading to negative effects on their overall health and well-being. These impairments can have a significant impact on older adults’ perceptions of their health condition (Solheim et al., 2011). Notably, SRH was also used as a predictor of cognitive decline (Aguiñaga et al., 2023). Previous studies found that older adults with good and stable changes in SRH over 8 years had the slowest rate of memory decline (Bendayan et al., 2017). An American cohort study found that poorer SRH in midlife was related to a higher likelihood of cognitive impairment 18 years later (Wu et al., 2022). However, some research also exists that suggests that cognitive impairment is not directly related to SRH unless sensory impairments are present at the same time (Liu et al., 2016). Given that older adults with hearing loss may experience a decline in cognitive function due to poor SRH, we hypothesized that SRH may be a mediating variable between hearing loss and cognitive function (H1).

Depression and anxiety are common in patients with cognitive impairment. The hazard ratios (HR) for conversion to dementia in older adults with MCI combined with anxiety or depression were 1.18 and 4.80, respectively, compared with older adults with MIC alone (Makizako et al., 2016; Li and Li, 2018). In addition, the link between hearing loss and mental health is clear. An American study found anxiety and depression in approximately one-fifth of older adults with hearing loss (Simning et al., 2019). However, existing studies have some limitations. When studying the connection between hearing loss and cognitive function, the majority of studies have mostly focused on the influence of depression rather than anxiety. For instance, a study of 8,094 older adults aged 65 years and older in China found that depression partially mediated the relationship between hearing loss and cognitive function, whereas the indirect effect of depression accounted for only 5.07% of the total effect (Cao et al., 2023), suggesting the existence of other important mediating variables. Depression and anxiety often coexist and interact with each other (Jacobson and Newman, 2017). Data suggest that approximately 85% of depressed patients experience significant symptoms of anxiety, and up to 90% of patients with anxiety have comorbid depressive symptoms, making it difficult to distinguish them (Hunt et al., 2002). This study fully considered the comorbidity of anxiety and depression. We employed latent variables, and the inclusion of both anxiety and depression, two observable factors, may provide a fuller picture of an individual’s mental health. Therefore, we further hypothesized that depressive anxiety symptoms may mediate the relationship between hearing loss and cognitive function (H2).

Previous evidence suggests that SRH may have a complex bidirectional relationship with depressive anxiety symptoms. SRH can be used in general practice to further assess individuals with depression and anxiety (Östberg and Nordin, 2022). In turn, depression and anxiety are independent predictors of poor SRH over 7 years (Rouch et al., 2014). Previous studies have looked into the link between SRH and depressive anxiety symptoms and cognitive function in the fields of genetics, pathology, and epidemiology. For example, behavioral genetics studies suggest that the association between SRH and spatial reasoning and perceptual speed is mediated by genetic and nonshared environmental factors in individuals aged up to 67 years, whereas the association between SRH and overall cognitive function after age 67 is entirely due to genetic factors (Svedberg et al., 2009). Neuropathological studies have suggested that anxiety or depression interact synergistically with increased brain amyloid deposition to increase the risk of MCI (Pink et al., 2022). Evidence from epidemiological studies suggests that patients with clinical depression and comorbid anxiety had deficits in multiple domains of cognitive functioning compared with those with clinical depression alone (Liu et al., 2020). Furthermore, compared with older adults with normal hearing, those with hearing loss had a 1.67-fold and 1.35-fold increased risk of poor SRH and depression, respectively (Yu and Liljas, 2019), and depressive anxiety symptoms is apt to occur in older adults with subpar SRH. However, the mechanisms by which SRH and depressive anxiety symptoms synergistically affect the link between hearing loss and cognitive function remain unclear. Considering SRH and depressive anxiety symptoms may be reciprocal, we employed two serial mediation models to test all combinations and analyze the mediating roles of SRH and depressive anxiety symptoms in the causal chain in specific flows. Therefore, we hypothesized that SRH and depressive anxiety symptoms serve as serial mediators in the link between hearing loss and cognitive function (H3).

**Figure 1:**
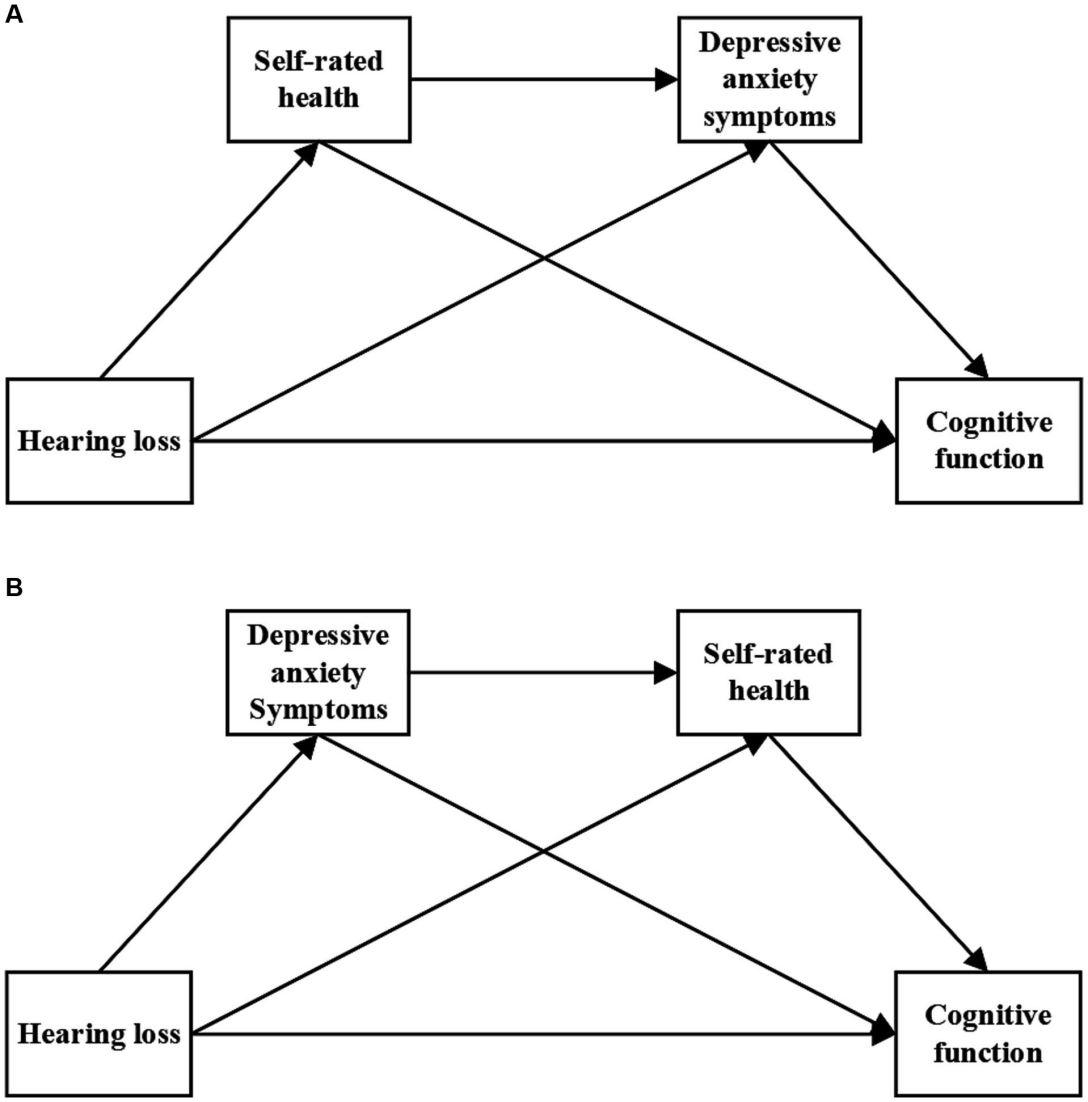
Hypothetical model. Panel **(A)** represents the serial mediation model 1 and Panel **(B)** represents the serial mediation model 2.

To further investigate the relationship between hearing loss, SRH, depressive anxiety symptoms, and cognitive function, the current study proposed two serial mediation models (Figure 1). The hypothesized model can be used to elucidate the underlying mechanisms and enrich the prevention policy of cognitive impairment in older adults, which has important implications for public health.

## 2 Methods

### 2.1 Study design and participants

A cross-sectional study with a stratified whole-group random sampling method was conducted in Urumqi, Xinjiang, China. First, the nine municipal districts of Urumqi were divided into district stratification, and three municipal districts were randomly selected using the random number table method. Next, one community in each municipal district was randomly selected using the same method. Three communities were finally selected as representative residents in this study. Participants were recruited from community health centers between January and July 2022, and face-to-face interviews with participants and on-site data collection were conducted. As a result of the aging process, it is possible that older individuals experiencing hearing loss may encounter challenges in accurately responding to inquiries owing to their diminished auditory capabilities. Thus, all investigators were professionally trained. When participants had hearing difficulties, investigators minimized the effect of hearing difficulties on question answering by slowing down the speed of speech, increasing the volume of the voice, or using the text form so that the participants could understand the items of the questionnaires clearly and answer them accordingly. In addition, to avoid language and cultural differences, the investigators included health workers and medical students who were familiar with the ethnic languages. Inclusion criteria were as follows: (1) current patients aged ≥65 years; (2) living in a community for >6 months; (3) volunteering to participate and providing informed consent for this study; and (4) clear consciousness and no language impairment. Exclusion criteria were as follows: (1) patients with physician-diagnosed dementia, mental or severe physical illness that prevents them from performing cognitive assessment tests; (2) hospitalized and elderly patients who are bedridden for long periods.

The N:q ratio is considered a simple and reliable rule to estimate the minimum sample size of a structural equation model because it takes into account the complexity of the model to be estimated, in which N is the number of samples and q is the parameter to be estimated in the model. Bentler and Chou (1987) state that a ratio of at least 5:1 between the sample size and the parameter being estimated is required to ensure that the estimate of the parameter is plausible, and a ratio of at least 10:1 is required to ensure the validity of the significance test (Bentler and Chou, 1987). Jackson (2003) suggests that 20:1 is a more desirable ratio (Jackson, 2003). In this study we used a ratio of 20:1 to determine the minimum sample size, where 22 parameters (coefficients of 6 paths, factor loadings between 5 observed and latent variables, measurement errors of 9 observable variables, and structural errors of 2 endogenous latent variables) needed to be estimated in this study, and a sample size of at least 440 is recommended. Recruiting a total of 646 participants, of whom 12 had dementia, 2 had missing data on both SRH and hearing function, 3 had missing data on SRH, 1 had missing data on hearing function, and 4 had missing data on cognitive function. By including 624 participants, the ultimate study sample met the minimum requirements for sample size.

### 2.2 Ethical statement

The Medical Ethics Committee of the First Affiliated Hospital of Xinjiang Medical University approved this study (Ethics Approval No. K202009-05). All participants provided written informed consent.

### 2.3 Measures

#### 2.3.1 Hearing loss

Hearing loss was measured by self-report (Valete-Rosalino and Rozenfeld, 2005). Participants examined their hearing status by answering the following question: “Do you have any difficulty hearing without the use of hearing aids?” “Difficulty” was coded as having a hearing loss, while “no difficulty” indicated no hearing loss.

#### 2.3.2 SRH

SRH was determined based on the following question: “How do you think your health is? Is it very poor, poor, fair, good, or excellent?” SRH was measured using a 5-point scale, with 1 being very poor and 5 being excellent. The higher the score, the better the SRH.

#### 2.3.3 Depressive anxiety symptoms

##### 2.3.3.1 Depression

The 15-item Geriatric Depression Scale (GDS-15) is a 15-item assessment scale designed by Sheikh and Yesavage based on the 30-item Geriatric Depression Scale (GDS-30) developed by Brink and Yesavage in 1982 (Yesavage et al., 1982; Sheikh and Yesavage, 1986). The GDS-15 scale assesses older adults’ negative thoughts, low mood, reduced activity level, and negative perceptions of their present and future lives. Participants answered questions based on how they felt the past week, with a total score ranging from 0 to 15, with higher scores indicating greater depressive symptoms, whereas scores ≥8 indicate depressive symptoms. The reliability and validity of the GDS-15 scale as a depression screening tool were demonstrated in a community-dwelling Asian elderly population (Nyunt et al., 2009). In this study, the Cronbach’s alpha coefficient for the scale was 0.716.

##### 2.3.3.2 Anxiety

The 7-item Generalized Anxiety Disorder scale (GAD-7) was used to assess anxiety symptoms (Spitzer et al., 2006). It consists of seven items that are used to assess how frequently participants experienced each symptom in the previous two weeks, and each item is scored from 0 to 3, equivalent to “none,” “a few days,” “over 50 % of the time,” and “almost every day.” The overall GAD-7 scale score has a range of 0 to 21, with a score of 5 and above indicating the presence of anxiety symptoms. In this study, the Cronbach’s alpha coefficient for the scale was 0.910.

#### 2.3.4 Cognitive function

Cognitive function was measured through the Mini-Mental State Examination (MMSE) (Folstein et al., 1975). The scale is one of the most extensively used tools in cognitive impairment evaluation, with its ratings being impacted by literacy. It contains 30 items with five dimensions: orientation (10 items), memory (3 items), attention and calculation (5 items), recall (3 items), and language capacity (9 items). The maximum score on the MMSE is 30, with lower scores indicating poorer cognitive function. The division boundaries for cognitive impairment were: illiteracy score ≤ 17, primary school score ≤ 20, and junior high school and above score ≤ 24. In this study, the Cronbach’s alpha coefficient for the scale was 0.910.

#### 2.3.5 Covariates

Participants’ demographic information, lifestyle, fall history in the previous year, nutritional status, and somatic function were collected during the baseline study. Demographic information included age, gender, ethnicity, education level, living alone, and monthly household income. Lifestyle included a history of smoking and drinking. Fall history was determined by self-report. The Mini Nutritional Assessment Short Form (MNA-SF) was used to assess nutritional status; an MNA-SF score of ≤11 was defined as malnutrition (Rubenstein et al., 2001). For the evaluation of somatic function, the instrumental activities of daily living (IADL) scale was used, and an IADL score of <8 was defined as IADL impairment (Lawton and Brody, 1969).

### 2.4 Statistical analysis

For data processing and statistical analysis, IBM SPSS 27.0 was used. Continuous variables were assessed for normality using the Kolmogorov-Sminov test. Normally distributed data are expressed as mean ± standard deviation(SD), whereas non-normally distributed data are expressed as median with an interquartile range of 25–75% in parentheses. Categorical variables were described using the number of cases and percentages. Chi-square tests and Fisher’s exact tests were used to compare sociodemographic and health-related characteristics between participants with and without cognitive impairment. Differences in MMSE scores across sociodemographic and health-related characteristics were compared using the Mann–Whitney test and the Kruskal-Wallis test. Spearman’s correlations were conducted to test the relationships between hearing loss, SRH scores, depressive symptom scores, anxiety symptom scores, and cognitive function scores. Statistical significance was defined as a value of p of <0.05.

IBM AMOS version 26.0 was used to construct the structural equation model, and since the hearing function is a dichotomous variable, we processed it as a dummy variable before incorporating it into the study model, used the maximum likelihood method for parameter estimation, and evaluated the model fit using the goodness-of-fit test. The following criteria were used to judge the goodness of fit of the model: the chi-squared divided by the degrees of freedom (χ^2^/df) ratio was less than 3, Goodness of Fit Index (GFI), Adjusted Goodness of Fit Index (AGFI), Comparative Fit Index (CFI), Normed Fit Index (NFI), and Tucker-Lewis Index (TLI) were greater than 0.90, and the root mean square error of approximation (RMSEA) was less than 0.08; The stability of the mediating effects and parameter estimates were examined using the Bootstrap method (Barber and Thompson, 2000). Bootstrapping is a type of repeated sampling in which a random sample of the same size is drawn with a return, and does not require the assumption of normality of the sampling distribution. We used 5,000 bootstrap resamples to calculate 95% bias-corrected confidence intervals (Preacher and Hayes, 2008). If the upper and lower limits did not include 0, it was statistically significant.

Robustness tests were conducted to determine the validity of the study model. The first method is, the alternative modeling method. Model 4 of the SPSS Macro Process was used to test for mediating effects (Hayes and Preacher, 2014), and variables that differed significantly in the chi-square test and Fisher’s exact test were used as control variables. The second method, changing the sample composition. Expanding the sample size fills in missing data values through multiple interpolations. Reducing the sample size tested the male sample by stratifying for gender.

### 2.5 Common method biases

Data collected through participants’ self-reports may be subject to common method bias, and this bias may lead to misinterpretation of the study results. To rule out common method bias, Harman’s one-factor test was utilized (Podsakoff et al., 2003). This revealed that the amount of variance explained by the first common factor accounted for was 20.89% (<40%), indicating that there was no substantial common method bias in the study’s data.

## 3 Results

### 3.1 Baseline characteristics

Participants were 624 community-dwelling older adults with a mean age of 72.42 ± 6.07 years were included, of which 151 (24.2%) had cognitive impairment and 249 (39.9%) had hearing loss. In addition, only 9 of 249 older adults with hearing loss wore hearing aids (3.61%), and 33.7% of all older adults with hearing loss had cognitive impairment. Sociodemographic and health-related characteristics of participants grouped by cognitive function are shown in Table 1. We found significant differences between the different cognitive function groups in terms of gender, age, number of falls, nutritional status, depressive symptoms, anxiety symptoms, SRH, and hearing function (*p* < 0.05), whereas the differences were not statistically significant regarding ethnicity, education level, living alone, monthly household income, smoking history, drinking history, and IADL condition (*p* > 0.05). A comparison of the MMSE scores of older adults with diverse characteristics revealed that differences in MMSE scores were statistically significant in terms of age, education level, living alone, monthly household income, nutritional status, number of falls in the previous year, hearing function, depressive symptoms, anxiety symptoms, and SRH (*p* < 0.05), whereas the differences were not statistically significant regarding gender, ethnicity, smoking history, drinking history, and IADL condition (*p* > 0.05) (Supplementary Table S1).

**Table 1:** Characteristics of study participants stratified by cognitive function (n = 624).

Variable	Categories	Cognitive function		*χ2*	*p*-value
		Normal *N* = 473 n (weighted %)	Impaired *N* = 151 n (weighted %)		
Gender	Male	189 (40.0%)	79 (52.3%)	7.137	0.008
	Female	284 (60.0%)	72 (47.7%)		
Age(years)	65–74	331 (70.0%)	80 (53.0%)	22.644	<0.001
	75–84	131 (27.7%)	57 (37.0%)		
	≥85	11 (2.3%)	14 (9.3%)		
Ethnicity	Han	392 (82.9%)	122 (80.8%)	0.341	0.559
	Ethnic minority	81 (17.1%)	29 (19.2%)		
Education level	Illiteracy	75 (15.9%)	24 (15.9%)	9.095	0.059
	Elementary school	145 (30.7%)	31 (20.5%)		
	Middle school	142 (30.0%)	49 (32.5%)		
	Senior high school	73 (15.4%)	36 (23.8%)		
	College and above	38 (8.0%)	11 (7.3%)		
Living alone	Yes	76 (16.1%)	34 (22.5%)	3.287	0.070
	No	397 (83.9%)	117 (77.5%)		
Monthly household income (CNY)	<1,000	25 (5.3%)	5 (3.3%)	6.656	0.155
	1,000–2,999	107 (22.6%)	33 (21.9%)		
	3,000–4,999	149 (31.5%)	40 (26.5%)		
	5,000–9,999	154 (32.6%)	65 (43.0%)		
	≥10,000	38 (8.0%)	8 (5.3%)		
Smoking history	Yes	58 (12.3%)	17 (11.3%)	0.109	0.741
	No	415 (87.7%)	134 (88.7%)		
Drinking history	Yes	84 (17.8%)	20 (13.2%)	1.679	0.195
	No	389 (82.8%)	131 (86.8%)		
Nutritional status	Malnutrition	32 (6.8%)	23 (15.2%)	10.208	0.001
	Normal	441 (93.2%)	128 (84.8%)		
IADL condition	Impaired	21 (4.4%)	3 (2.0%)	1.862	0.172
	Not impaired	452 (95.6%)	148 (98.0%)		
Number of falls	Never	389 (82.2%)	115 (76.2%)	8.653	0.013
	1–3	66 (14.0%)	21 (13.9%)		
	≥4	18 (3.8%)	15 (9.9%)		
Depressive symptoms	Yes	11 (2.3%)	11 (7.3%)	8.276	0.004
	No	462 (97.7%)	140 (92.7%)		
Anxiety symptoms	Yes	74 (15.6%)	44 (29.1%)	13.592	<0.001
	No	399 (84.4%)	107 (70.9%)		
SRH	Very poor	1 (0.2%)	0 (0.0%)	26.286	<0.001
	Poor	31 (6.6%)	30 (19.9%)		
	Fair	162 (34.2%)	51 (33.8%)		
	Good	212 (44.8%)	59 (39.1%)		
	Excellent	67 (14.2%)	11 (7.3%)		
Hearing function	Normal	308 (65.1%)	67 (44.4%)	20.542	<0.001
	Loss	165 (34.9%)	84 (55.6%)		

CNY, Chinese Yuan. IADL, Instrumental Activity of Daily Living; SRH, Self-rated health.

### 3.2 Correlation analysis of the main study variables

Descriptive statistics and Spearman’s correlations for the main study variables are shown in Table 2. Hearing loss was found to have a positive connection with anxiety (*r* = 0.167, *p* < 0.01) and depression (*r* = 0.195, *p* < 0.01). SRH (*r* = −0.350, *p* < 0.01) and cognitive function (*r* = −0.164, *p* < 0.01) were both adversely linked with hearing loss.

**Table 2:** Spearman correlation analysis between the main study variables.

Variable	M ± SD	Hearing loss	Depression	Anxiety	SRH	Cognitive function
Hearing loss	0.40 ± 0.49	1				
Depression	2.16 ± 2.13	0.195^**^	1			
Anxiety	2.31 ± 3.20	0.167^**^	0.414^**^	1		
SRH	3.58 ± 0.84	−0.350^**^	−0.305^**^	−0.274^**^	1	
Cognitive function	24.68 ± 5.41	−0.164^**^	−0.106^**^	−0.164^**^	0.212^**^	1

SRH, Self-rated health; ^**^Correlation is significant at the 0.01 level (two-tailed).

### 3.3 Study model testing

**Figure 2:**
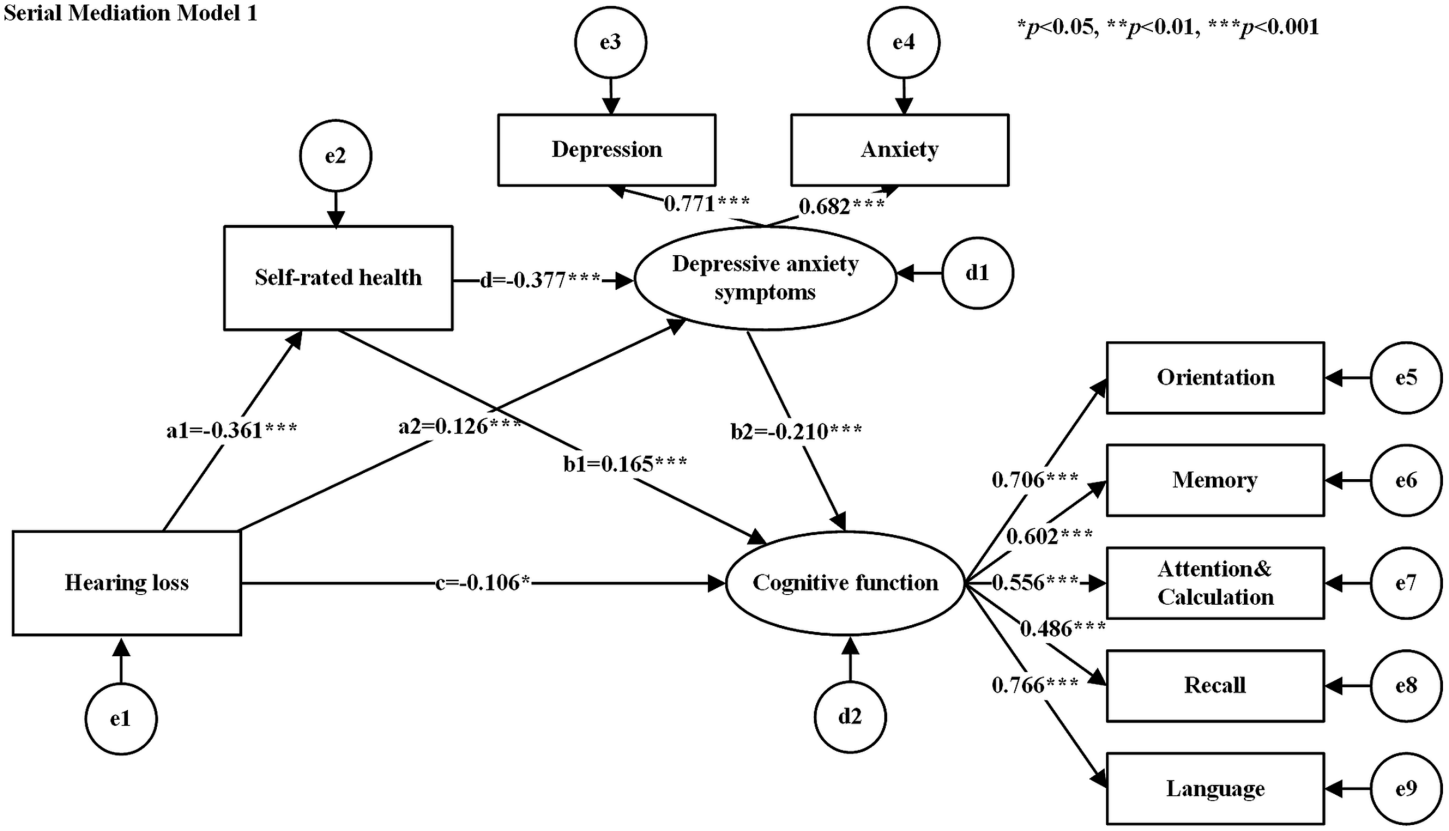
Serial mediation model 1. Rectangles for observed variables, ellipses for latent variables. e1−e9 denote measurement errors of the corresponding observed variables and d1−d2 denote structural errors of the corresponding endogenous variables. The values attached to the arrows are the direct effects of normalization. The numbers attached to the arrows between the latent and observed variables are the standardized factor loading values. Serial mediation model 2 same as above.

**Figure 3:**
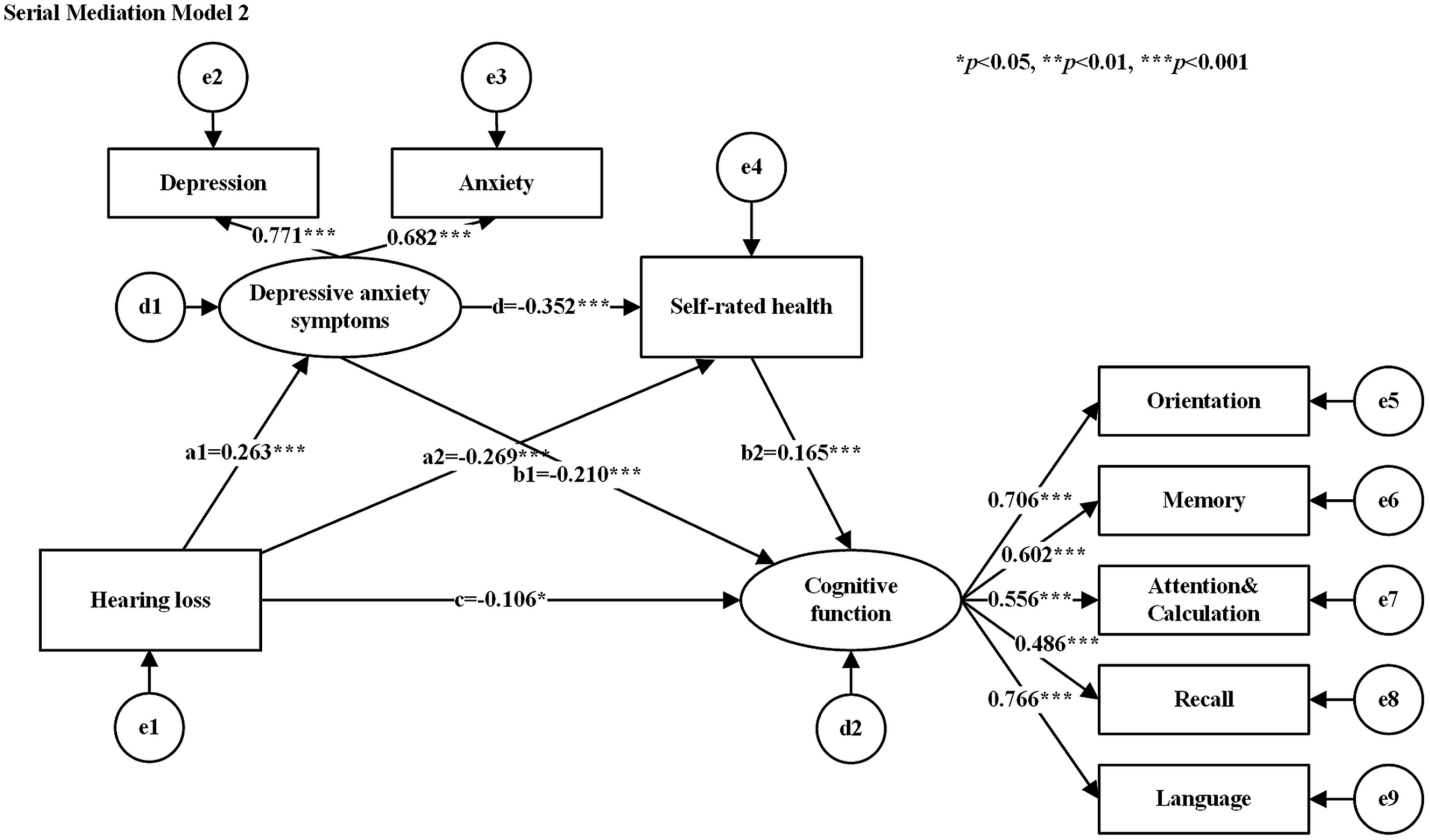
Serial mediation model 2.

The hypothesized models were tested for goodness-of-fit, and the results of the goodness-of-fit tests for the serial mediation model 1 and serial mediation model 2 were consistent with the following results: χ^2^/df = 2.662, GFI = 0.979, AGFI = 0.958, CFI = 0.967, NFI = 0.949, TLI = 0.948, and RMSEA = 0.052, all of which met their respective criteria. Table 3 shows the standardized estimates of the paths in each serial mediation model. The results of the path analyses showed that hearing loss had a negative effect on SRH, cognitive function, and a positive effect on depressive anxiety symptoms. SRH had a negative effect on depressive anxiety symptoms (See Figure 2 for serial mediation model 1 and Figure 3 for serial mediation model 2).

**Table 3:** Path coefficients of the study model.

		Path		Standardized Regression Weights	SE	CR	*P*
Serial Mediation Model 1							
c	HL	→	*CF*	−0.106	0.118	−2.263	0.024
a1	HL	→	SRH	−0.361	0.064	−9.671	<0.001
a2	HL	→	DAS	0.126	0.217	2.596	0.009
b1	SRH	→	*CF*	0.165	0.077	3.175	0.001
b2	NE	→	*CF*	−0.210	0.035	−3.432	<0.001
d	SRH	→	DAS	−0.377	0.144	−6.844	<0.001
Serial Mediation Model 2							
c	HL	→	*CF*	−0.106	0.118	−2.263	0.024
a1	HL	→	DAS	0.263	0.225	5.201	<0.001
a2	HL	→	SRH	−0.269	0.065	−7.074	<0.001
b1	DAS	→	*CF*	−0.210	0.035	−3.432	<0.001
b2	SRH	→	*CF*	0.165	0.077	3.175	0.001
d	DAS	→	SRH	−0.352	0.019	−7.075	<0.001

HL, Hearing loss; SRH, Self-rated health; DAS, Depressive anxiety symptoms; CF, Cognitive function; SE, Standard error; CR, Critical ratio.

### 3.4 Serial mediation model

The study examined the relationship between SRH, depressive anxiety symptoms, and cognitive function by analyzing two different causal orders. Table 4 shows the bootstrap results for indirect effects. The results of the serial mediation analysis showed that the total indirect effect of SRH and depressive anxiety symptoms was −0.115 (95% CI: −0.168 to −0.070), and the total effect of the model was −0.221 (95% CI: −0.307 to −0.132), with the total indirect effect accounting for 52.04% of the total effect of the model. In serial mediation model 1, the indirect effect path (HL → SRH → DAS → *CF*) had an indirect effect of −0.029 (SE = 0.013, 95%: −0.058 to −0.007). In the serial mediation model 2, the indirect effect paths (HL → DAS → SRH → *CF*) had an indirect effect of −0.016 (SE = 0.006, 95%: −0.030 to −0.005). In the relationship between hearing loss and cognitive functions, SRH and depressive anxiety symptoms co-played a serial mediating role. This suggests that loss of hearing function leads to an increased risk of poor SRH or depressive anxiety symptoms, which in turn leads to a decline in cognitive function.

**Table 4:** Comparisons of the bootstrap results of the serial mediation models 1 and 2 (standardized coefficients).

Path	Estimate	SE	*p*	95%Bias-corrected confidence intervals		Percentage of Total Effects (%)
				Lower	Upper	
Total effect	−0.221	0.045	<0.001	−0.307	−0.132	100
Direct effect	−0.106	0.047	0.021	−0.201	−0.016	47.96
Total indirect effect	−0.115	0.025	<0.001	−0.168	−0.07	52.04
Serial Mediation Model 1						
Indirect effect (HL → SRH → *CF*)	−0.059	0.022	0.005	−0.103	−0.018	26.70
Indirect effect (HL → DAS → *CF*)	−0.027	0.015	0.013	−0.066	−0.005	12.22
Indirect effect (HL → SRH → DAS → *CF*)	−0.029	0.013	0.009	−0.058	−0.007	13.12
Serial Mediation Model 2						
Indirect effect (HL → DAS → *CF*)	−0.055	0.026	0.008	−0.155	−0.013	24.89
Indirect effect (HL → SRH → *CF*)	−0.044	0.017	0.005	−0.080	−0.013	19.91
Indirect effect (HL → DAS → SRH → *CF*)	−0.016	0.006	0.003	−0.030	−0.005	7.24

HL, Hearing loss; SRH, Self-rated health; DAS, Depressive anxiety symptoms; CF, Cognitive function; SE, Standard error.

### 3.5 Robustness check

First, the alternative modeling approach. The independent mediating roles of SRH, depression, and anxiety between hearing loss and cognitive functioning were tested, controlling for gender, age, number of falls, and nutritional status variables. The results showed that the mediating effects of SRH, depression, and anxiety were all held after controlling for confounders (see Supplementary Table S2 and Figure 1). Second, the sample composition was changed. The model was retested using the new sample after filling in missing values (sample size = 634) and the male sample (sample size = 268). The results showed that the model fit was more favorable to the data, and the results of the mediation analyses were generally consistent with the original model (see Supplementary Table S3).

## 4 Discussion

The present study found a 24.2% prevalence of cognitive impairment in older adults from the Xinjiang community in northwestern China, which was somewhat higher than the general prevalence of 22.4% of cognitive impairment in China (Qin et al., 2022) and higher than the prevalence of 6.3% in the UK (Richardson et al., 2019). We also noted self-reported hearing loss among 39.9% of older adults, which was much higher than the 9.7% in Japan (Kawaguchi et al., 2023) and 23.2% in Korea (Lee and Chung, 2023). Given the high prevalence of cognitive impairment and hearing loss among community-dwelling older adults in northwestern China, local health organizations and relevant authorities should put a high premium on this situation. Furthermore, we found that hearing loss not only affects cognitive function directly but also indirectly through SRH and depressive anxiety symptoms. Moreover, SRH and depressive anxiety symptoms as mediators had a total mediating effect of 52.04%, greater than the direct effect of hearing loss on cognitive function, suggesting that our mediators are critical in explaining how hearing loss relates to cognitive function.

### 4.1 Direct effect of hearing loss on cognitive function

Our findings showed that hearing loss significantly and negatively affected cognitive function in older adults. We also found that 33.7% of older adults with hearing loss suffered from cognitive impairment. This result indicates that hearing loss poses a serious threat to the cognitive health of older adults. Multiple hypotheses have been used to explain the mechanisms linking hearing loss and cognitive decline. For example, the common etiology hypothesis suggests that the association between hearing loss and cognitive decline may be due to common neurodegenerative disorders and there is no clear causal relationship between the two (Uchida et al., 2019). According to the sensory deprivation hypothesis, auditory decline leads to a reduction in the transmission of auditory information to the brain and a long-term lack of a sufficient amount of perceptual stimulation, causing atrophy in areas of the auditory system, resulting in the onset of cognitive decline (Slade et al., 2020). According to the information degradation hypothesis, patients with hearing loss tend to listen hard, which can overconsume the brain’s cognitive resources to offset the absence of auditory information, and in the long term, cognitive resources will be over-utilized, leading to cognitive decline (Powell et al., 2021). Although the exact underlying mechanism between hearing loss and cognitive decline remains unclear, current research suggests that restoring auditory input improves auditory neural function and cognitive function in hearing loss patients (Karawani et al., 2018). Hearing loss interventions can prevent or delay 8% of dementia (Livingston et al., 2020). Therefore, the management of hearing in older adults should be enhanced to improve hearing function and reduce cognitive deterioration and other adverse effects.

### 4.2 Mediating role of SRH

To the best of our knowledge, few studies to date have used SRH as a mediator to investigate the relationship between hearing loss and cognitive function. Our study demonstrated that hearing loss can indirectly affect cognitive function in older adults through SRH. Although there is a high prevalence of poor SRH in older adults with hearing loss, the underlying mechanisms remain unclear. Most studies support that the nature of hearing loss (congenital or acquired) and the degree to which hearing loss causes limitations in daily activities affect SRH (Anderle et al., 2023). Hearing loss in older adults not only affects their daily communication and interactions but also leads to other health problems such as impaired lower limb function, frailty syndrome, and IADL disability (Yévenes-Briones et al., 2021). Thus, older people with hearing loss may perceive significant changes in their lifestyle, activities, and social life due to physical limitations, and they may be aware of their health decline compared to their past or peers, thus making a poor evaluation of their health (Pinto et al., 2016). Hogan et al. (2015) argues that when an individual’s coping mechanisms are not well-suited to the demands of their environment, they are more likely to experience increased stress and diminished well-being, ultimately leading to a lower SRH (Hogan et al., 2015). Whitmore et al.’s (2023) study suggests that SRH may initially decrease when individuals experience a disease adversity, such as a new diagnosis. However, as resources are activated and older adults recover or adapt to adversity, SRH may return to its original levels or even increase Furthermore, Whitmore’s study indicates that individuals with congenital hearing impairment are not necessarily associated with poor SRH. This is because they develop alternative communication mechanisms apart from speaking and hearing. Moreover, after receiving hearing aids and/or rehabilitation, they develop coping strategies, such as sign language and oral-facial reading, to integrate into the community (Anderle et al., 2023). All of this evidence suggests that individuals who are unable to cope with the changes brought about by hearing loss and lack appropriate coping strategies, such as seeking professional help or using hearing aids, are more likely to experience poor SRH. Working memory refers to an individual’s ability to temporarily store and manipulate information while performing a cognitive task, and a poor SRH is associated with poorer overall cognition and working memory, which leads to decreased cognitive function (Aguiñaga et al., 2023). Thus, more emphasis should be placed on improving the poor SRH in older adults, which could be a novel approach to preventing or delaying cognitive decline.

### 4.3 Mediating role of depressive anxiety symptoms

Results from our study imply that older adults suffering from hearing loss are more likely to experience depression and anxiety, which raises the likelihood of cognitive deterioration. Hearing loss has historically been related to poor mental health (Bigelow et al., 2020). 1hearing loss and depressive anxiety symptoms. For instance, reduced auditory input leads to dysfunctional emotional processing circuits that are dysfunctional key limbic structures responsible for emotion and behavior, which can lead to impaired perception and misclassification of emotional responses (Zinchenko et al., 2018). Decreased amygdala and hippocampal responsivity to emotional sounds in patients with hearing loss is another potential mechanism, which can contribute to the occurrence of depression (Husain et al., 2014). Disrupted connections in the amygdala also exacerbate anxiety associated with hearing loss (Tang et al., 2020). Behavioral mechanisms have also been used to explain the relationship between hearing loss and depressive anxiety symptoms. Due to the negative impacts of hearing loss, such as social isolation, loneliness, and limited mobility, it can raise the risk of depressive anxiety symptoms (Sharma et al., 2021). Evidence suggests that anxiety and depression have severely adverse consequences on the transient memory domain of cognitive capability in patients with hearing loss, thereby accelerating cognitive decline (Andries et al., 2023). Additionally, anxiety and depression accelerate the rate of atrophy in the frontal and temporal lobes of the brain, respectively, facilitating the progression of MCI toward Alzheimer’s disease (Mah et al., 2015; Sacuiu et al., 2016). Thus, reducing anxiety and depressive symptoms in older adults with hearing loss possibly protects their cognitive function to some extent, which warrants further exploration in the future.

### 4.4 Serial mediation effect of SRH and depressive anxiety symptoms

A series of mediating effects of SRH and depressive anxiety symptoms provide new perspectives on the mechanisms through which hearing loss affects cognitive function. In serial mediation model 1, hearing loss initially caused poor SRH and subsequently increased the risk of depressive anxiety symptoms, which may have led to a more rapid cognitive decline. In addition to the high burden of hearing loss and its adverse influence on the quality of life, hearing loss affects patients’ SRH and psychosocial (Jayakody et al., 2022). Patients with hearing loss may have poor SRH due to limited social participation and disability in daily activities in the absence of hearing aids (Anderle et al., 2021). A Brazilian study found that the odds of reporting poor SRH in peers who perceived hearing loss as a health problem were 3.72 times higher compared with peers who failed to perceive hearing loss as a health problem (Guia et al., 2018). Poor SRH can lead to a lack of perceived self-worth and meaning in an individual’s life (Vogel et al., 2021). Therefore, patients with hearing loss are more likely to exhibit ‘disengaged coping’ to escape the stress and exhaustion related to social interactions, as well as the embarrassment of displaying hearing difficulties before other people, which implies avoiding addressing hearing loss by opting out or withdrawing, for instance, withdrawing from social gatherings or pretending to hear during conversations (Heffernan et al., 2016). This negative coping can exacerbate mental tension, undermine mental health, and lead to the onset of anxiety and depression. Depression and anxiety are clinical markers that can help identify early signs of cognitive decline (Perin et al., 2022).

In serial mediation model 2, hearing loss initially leads to depressive anxiety symptoms and subsequently increases the risk of poor SRH, which can lead to a decline in cognitive function. If individuals with hearing loss adopt appropriate coping mechanisms (e.g., care-seeking behaviors, hearing rehabilitation training, wearing hearing aids, and seeking social support), the negative psychosocial and health consequences of hearing loss can be mitigated (Wells et al., 2020). In this case, hearing loss patients have a low risk of depression and anxiety and tend to make a positive assessment of their health. Moreover, individuals with good SRH perform better in executive function, working memory, and global cognition than individuals with poor or fair SRH (Aguiñaga et al., 2023). This study found that SRH and depressive anxiety symptoms are interrelated, regardless of the direction in which they flow between hearing loss and cognitive function, both of which have a serial mediation effect, shedding light on the mechanisms through which hearing loss impacts cognition. These findings suggest that older individuals should prioritize not only the management of hearing loss but also the management of SRH and mental health.

### 4.5 Implications and limitations

This study innovatively used SRH and depressive anxiety symptoms as mediating variables and confirmed that both SRH and depressive anxiety symptoms partially mediated the relationship between hearing loss and cognitive function in community-dwelling older adults. These findings may help identify people at high risk of cognitive impairment and ultimately prevent and treat this disease through the management of SRH and depressive anxiety symptoms in older adults with hearing loss. According to the data, the prevalence of hearing loss among Chinese adults over 60 years old is 58.85%, but the rate of hearing aid acquisition is only 6.5% (Gong et al., 2018), which is lower than the 10% in Japan and 17.4% in South Korea (Moon et al., 2015; Sugiura et al., 2022). Reliable evidence suggests that the use of hearing aid devices in patients with hearing loss reduces the risk of cognitive decline by 19% (Yeo et al., 2023). In this study, only 3.61% of older adults with hearing loss had access to hearing aids, well below the national level and in other Asian countries. We suggest that older adults with hearing loss should be promptly identified and managed to improve the utilization of hearing aid devices. Future studies should also develop cognitive function assessment tools that are more applicable to older adults with hearing loss. Community and medical personnel should regularly assess SRH and the mental health of older adults, especially those with hearing loss, which may facilitate the early detection of cognitive problems. Previous research found that the number and quality of social networks can buffer the adverse impacts of poor SRH on mental health (Windsor et al., 2016), and therefore older adults should be encouraged to engage in more social group activities regularly to build a good social support system.

Although this study has some theoretical and practical implications, some limitations exist. First, this cross-sectional study did not fully elucidate the causal relationships among hearing loss, SRH, depressive anxiety symptoms, and cognitive function. Longitudinal studies with large sample sizes are needed to comprehensively explore the causal relationships among the investigated variables. Second, hearing loss was determined by self-report in this study. Although self-reported hearing loss is a brief and highly effective indicator in epidemiological studies, there is a need to jointly use self-reported hearing loss with hearing status measured by pure-tone audiometry for screening and research (Louw et al., 2018). Finally, the study variables in this study were surveyed by questionnaire and may be subject to reporting bias.

## 5 Conclusion

In summary, this study investigated the relationships among hearing loss, SRH, depressive anxiety symptoms, and cognitive function. The pathway analysis revealed that hearing loss can affect cognitive function directly as well as indirectly through the serial mediation effects of SRH and depressive anxiety symptoms. These findings may help to identify and manage cognitive problems in older adults on time. Additionally, focusing on enhanced management of hearing loss — complemented by the management of SRH and mental health — may be another effective public health strategy to protect cognitive function in older adults.

## Data availability statement

The datasets presented in this article are not readily available because the data are available from the corresponding author upon reasonable request. Requests to access the datasets should be directed to FC, 1152065261@qq.com.

## Ethics statement

The studies involving humans were approved by Medical Ethics Committee of the First Affiliated Hospital of Xinjiang Medical University (Ethics Approval No. K202009-05). The studies were conducted in accordance with the local legislation and institutional requirements. The participants provided their written informed consent to participate in this study.

## Author contributions

FC: Funding acquisition, Investigation, Supervision, Writing – review & editing. YC: Conceptualization, Formal analysis, Writing – original draft. XJ: Investigation, Visualization, Writing – review & editing. XL: Formal analysis, Writing – review & editing. HN: Methodology, Writing – review & editing. MH: Validation, Writing – review & editing. WJ: Writing – review & editing, Data curation. NZ: Writing – review & editing. HF: Conceptualization, Project administration, Supervision, Writing – review & editing. PY: Conceptualization, Funding acquisition, Investigation, Writing – review & editing.
