# Supplementary material for: Impact of hearing loss on cognitive function in community-dwelling older adults: serial mediation of self-rated health and depressive anxiety symptoms
Source: Front Aging Neurosci. 2023 Dec 14;15:1297622. doi: 10.3389/fnagi.2023.1297622 (PMC10753014; doi:10.3389/fnagi.2023.1297622)
Supplement: Supplementary file 1 [file Data_Sheet_1.docx]

***Supplementary Material***

**Impact of hearing loss on cognitive function in community-dwelling older adults: Serial mediation of self-rated health and depressive anxiety symptoms**

**Supplementary Table 1** Comparison of MMSE scores in older adults with different characteristics (n = 624).

| Variable | Categories | N (%) | Median MMSE  (P25, P75 ) | *z*/*H*-value | *p*-value |
| --- | --- | --- | --- | --- | --- |
|  |  |  |  |  |  |
| Gender | Male | 268(42.9) | 26.50(23.00, 28.75) | -1.121 | 0.262 |
|  | Female | 356(57.1) | 26.00(22.00, 28.00) |  |  |
| Age(years) | 65-74 | 411(65.9) | 27.00(24.00, 29.00) | 43.915 | <0.001 |
|  | 75-84 | 188(30.1) | 25.00(21.00, 28.00) |  |  |
|  | ≥85 | 25(4.0) | 22.00(8.00, 25.50) |  |  |
| Ethnicity | Han | 514(82.4) | 26.00(23.00,29.00) | -1.266 | 0.206 |
|  | Ethnic minority | 110(17.6) | 25.50(22.00,28.00) |  |  |
| Education level | Illiteracy | 99(15.9) | 22.00(18.00,24.00) | 137.877 | <0.001 |
|  | Elementary school | 176(28.2) | 25.00(22.00,28.00) |  |  |
|  | Middle school | 191(30.6) | 27.00(24.00,29.00) |  |  |
|  | Senior high school | 109(17.5) | 28.00(25.00,29.00) |  |  |
|  | College and above | 49(7.9) | 29.00(27.00,30.00) |  |  |
| Living alone | Yes | 110(17.6) | 25.00(22.00,28.00) | -2.641 | 0.008 |
|  | No | 514(82.4) | 26.00(23.00,29.00) |  |  |
| Monthly household income (CNY) | <1000 | 30(4.8) | 23.50(21.75,27.25) | 22.001 | <0.001 |
|  | 1000-2999 | 140(22.4) | 26.00(22.00,28.00) |  |  |
|  | 3000-4999 | 189(30.3) | 26.00(23.00,28.00) |  |  |
|  | 5000-9999 | 219(35.1) | 26.00(22.00,28.00) |  |  |
|  | ≥10000 | 46(7.4) | 28.00(26.00,29.25) |  |  |
| Smoking history | Yes | 75(12.0) | 26.00(22.00,28.00) | -0.133 | 0.894 |
|  | No | 549(88.0) | 26.00(23.00,28.00) |  |  |
| Drinking history | Yes | 104(16.7) | 26.00(22.00,28.00) | -0.879 | 0.379 |
|  | No | 520(83.3) | 26.00(23.00,28.00) |  |  |
| Nutritional status | Normal | 569(91.2) | 26.00(23.00,29.00) | -3.592 | <0.001 |
|  | Malnutrition | 55(8.8) | 24.00(17.00,27.00) |  |  |
| IADL condition | Not impaired | 600(96.15) | 26.00(23.00,28.00) | -1.010 | 0.312 |
|  | Impaired | 24(3.85) | 27.50(22.25,29.00) |  |  |
| Number of falls | Never | 504(80.8) | 26.00(23.00,29.00) | 12.409 | 0.002 |
|  | 1-3 | 87(13.9) | 26.00(22.00,28.00) |  |  |
|  | ≥4 | 33(5.3) | 25.00(16.50,27.00) |  |  |
| Depressive symptoms | Yes | 22(3.5) | 21.50(14.50,28.00) | -2.805 | 0.005 |
|  | No | 606(96.5) | 26.00(23.00,28.00) |  |  |
| Anxiety symptoms | Yes | 118(18.9) | 25.00(20.00,27.00) | -4.419 | <0.001 |
|  | No | 506(81.1) | 27.00(23.00,29.00) |  |  |
| SRH | Very poor | 1(0.2) | 29 | 38.500 | <0.001 |
|  | Poor | 61(9.8) | 22.00(15.50,26.50) |  |  |
|  | Fair | 213(34.1) | 26.00(22.00,28.00) |  |  |
|  | Good | 271(43.3) | 27.00(24.00,29.00) |  |  |
|  | Excellent | 78(12.5) | 26.50(23.00,29.00) |  |  |
| Hearing function | Normal | 375(60.1) | 27.00(23.00.29.00) | -4.089 | ＜0.001 |
|  | Loss | 249(39.9) | 25.00(21.00,28.00) |  |  |
| Cognitive function | Normal | 473(75.8) | 27.00(25.00,29.00) | -15.359 | <0.001 |
|  | Impairment | 151(24.2) | 20.00(15.00,24.00) |  |  |

Note: CNY, Chinese Yuan; IADL, Instrumental Activity of Daily Living; SRH, Self-rated health; MMSE, Minimum Mental State Examination.

| Independent Variable | Mediator | Dependent Variable | Effect of X on M | Effect of M on Y | Direct Effect | Indirect Effect | Total Effect |
| --- | --- | --- | --- | --- | --- | --- | --- |
| Hearing loss | SRH | Cognitive function | -0.5733  (SE=0.0640)*** | 0.8336  (SE=0.2569)** | -0.8593  (SE=0.4342)* | -0.4779  (95%CI: -0.8251 to -0.1720) | -1.3372  (SE=0.4116)** |
|  | Depression |  | 0.8979  (SE = 0.1720)*** | -0.2627  (SE = 0.0957)** | -1.1013  (SE=0.4184)** | -0.2359  (95%CI: -0.5048 to -0.0306) |  |
|  | Anxiety |  | 1.1175  (SE = 0.2586)*** | -0.2244  (SE = 0.0634)*** | -1.0864  (SE=0.4139)** | -0.2508  (95%CI:-0.4962 to -0.0656) |  |
| SRH: Self-rated health, SE: standard error, 95%CI: 95% confidence interval. | | | | | | | |
| Adjusted age, gender, number of falls, and nutritional status. | | | | | | | |
| * *p* <0.05; ** *p* < 0.01；****p* < 0.001 | | | | | | | |

**Supplementary Table 2:** A Mediating Role Models of Self-Rated Health, Depression, and Anxiety in the Relationship Between Hearing Loss and Cognitive Function.

**Supplementary Table 3:** Robustness checks for gender-stratified male samples and multiple interpolated samples.

|  | Effect | Estimate | SE | 95%CI (Lower, Upper) | Model fit |
| --- | --- | --- | --- | --- | --- |
| Male  sample  (N=268) | Total effect | -0.294 | 0.070 | -0.427 to -0.152 | χ^2^/df =1.801,  RMSEA=0.055,CFI=0.970,GFI=0.967,AGFI=0.936,  TLI=0.953,NFI=0.936 |
|  | Direct effect | -0.126 | 0.073 | -0.268 to -0.020 |  |
|  | Total indirect effect | -0.167 | 0.040 | -0.254 to -0.096 |  |
|  | HL→SRH→CF | -0.069 | 0.030 | -0.133 to -0.015 |  |
|  | HL→DAS→CF | -0.051 | 0.030 | -0.129 to -0.207 |  |
|  | HL→SRH→DAS→CF | -0.047 | 0.020 | -0.096 to -0.017 |  |
|  | Effect | Estimate | SE | 95%CI (Lower, Upper) | Model fit |
| Multiple interpolation sample  (N=634) | Total effect | -0.220 | 0.046 | -0.309 to -0.134 | χ^2^/df =2.986,  RMSEA=0.056,CFI=0.961,GFI=0.977,AGFI=0.954,  TLI=0.939,NFI=0.943 |
|  | Direct effect | -0.104 | 0.047 | -0.198 to -0.013 |  |
|  | Total indirect effect | -0.116 | 0.024 | -0.167 to -0.072 |  |
|  | HL→SRH→CF | -0.061 | 0.022 | -0.107 to -0.021 |  |
|  | HL→DAS→CF | -0.026 | 0.015 | -0.066 to -0.005 |  |
|  | HL→SRH→DAS→CF | -0.029 | 0.013 | -0.057 to -0.006 |  |
| HL: Hearing loss, SRH: Self-rated health, DAS, Depressive anxiety symptoms, CF: Cognitive function. | | | | | |
| SE: standard error, 95%CI: 95% confidence interval. | | | | | |


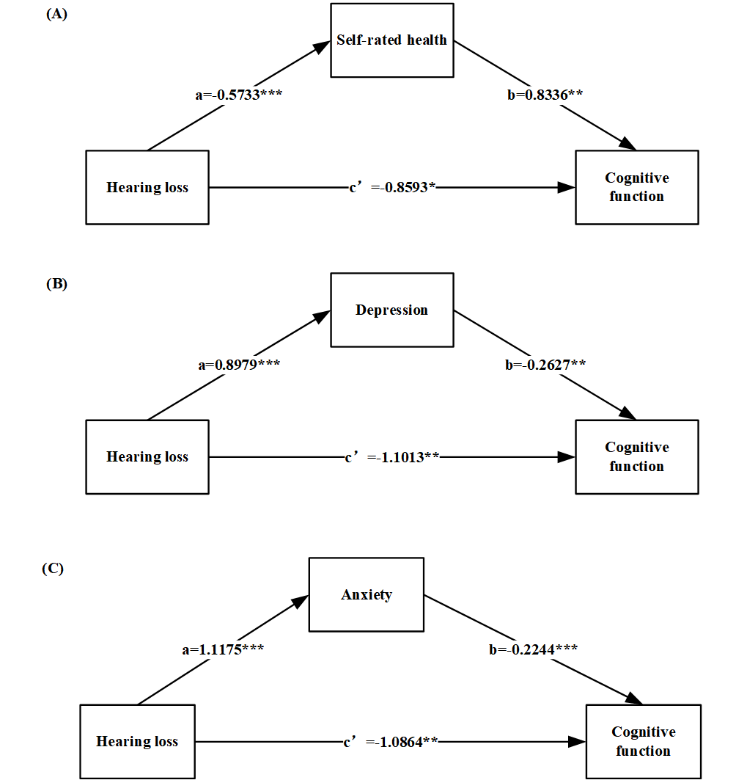


**Supplementary Figure 1.** Panel A, the mediating role of self-rated health between hearing loss and cognitive function; Panel B, the mediating role of depression between hearing loss and cognitive function; Panel C, the mediating role of anxiety between hearing loss and cognitive function. * *p* <0.05; ** *p* < 0.01；****p* < 0.001.
